# Estimating the risks of prehospital transfusion of D‐positive whole blood to trauma patients who are bleeding in England

**DOI:** 10.1111/vox.13249

**Published:** 2022-01-12

**Authors:** Rebecca Cardigan, Tom Latham, Anne Weaver, Mark Yazer, Laura Green

**Affiliations:** ^1^ Clinical Services NHS Blood and Transplant Cambridge UK; ^2^ Department of Haematology University of Cambridge Cambridge UK; ^3^ Clinical Services NHS Blood and Transplant London UK; ^4^ Department of Emergency Medicine, Barts Health NHS Trust London UK; ^5^ Department of Haematology, Barts Health NHS Trust London UK; ^6^ Department of Pathology University of Pittsburgh Pittsburgh Pennsylvania USA; ^7^ Blizard Institute Queen Mary University of London London UK

**Keywords:** HDFN, low‐titre group O whole blood, major haemorrhage, massive transfusion, red cells, trauma, whole blood

## Abstract

**Background and Objectives:**

D‐negative red cells are transfused to D‐negative females of childbearing potential (CBP) to prevent haemolytic disease of the foetus and newborn (HDFN). Transfusion of low‐titre group O whole blood (LTOWB) prehospital is gaining interest, to potentially improve clinical outcomes and for logistical benefits compared to standard of care. Enhanced donor selection requirements and reduced shelf‐life of LTOWB compared to red cells makes the provision of this product challenging.

**Materials and Methods:**

A universal policy change to the use of D‐positive LTOWB across England was modelled in terms of risk of three specific harms occurring: risk of haemolytic transfusion reaction now or in the future, and the risk of HDFN in future pregnancies for all recipients or D‐negative females of CBP.

**Results:**

The risk of any of the three harms occurring for all recipients was 1:14 × 10^3^ transfusions (credibility interval [CI] 56 × 10^2^–42 × 10^3^) while for females of CBP it was 1:520 transfusions (CI 250–1700). The latter was dominated by HDFN risk, which would be expected to occur once every 5.7 years (CI 2.6–22.5). We estimated that a survival benefit of ≥1% using LTOWB would result in more life‐years gained than lost if D‐positive units were transfused exclusively. These risks would be lower, if D‐positive blood were only transfused when D‐negative units are unavailable.

**Conclusion:**

These data suggest that the risk of transfusing RhD‐positive blood is low in the prehospital setting and must be balanced against its potential benefits.

**Highlights**

- We modelled the risk of harm that would be predicted from a policy change from transfusion of RhD‐negative to ‐positive whole blood in the prehospital setting.
- The risk of a haemolytic transfusion reaction due to an index D‐positive transfusion was 1:27,000 transfusions for all recipients and 1:6660 for D‐negative females under 50; one event occurring every 4 years and 65 years, respectively, in England.
- The main risk was that of haemolytic disease of the foetus and newborn, where for every 570 transfusions, one foetal death or disability would be predicted to occur, one event every 5.7 years in England.

## INTRODUCTION

UK guidelines recommend that for females of childbearing age, group O D‐negative red blood cells (RBC) should be administered if blood group is unknown, to prevent D alloimmunization, which can lead to haemolytic disease of the foetus and newborn (HDFN) in future pregnancies [1]. Group O D‐negative RBC are frequently transfused in the prehospital phase of resuscitation, as it is not possible to predict in advance if the patient is going to be a male or a woman of childbearing age or determine their D type. In the last decade our improved understanding of the biology of acute traumatic coagulopathy has resulted in the development of damage control resuscitation [2, 3, 4, 5], which advocates for the rapid and balanced administration of RBC, platelet and plasma as early as possible in the patient's resuscitation. This has improved outcomes for patients compared to the standard of care alone, especially in the prehospital phase of the resuscitation [6, 7, 8, 9]. The need to provide early haemostatic resuscitation with plasma has awakened significant interest in the reintroduction of low‐titre group O whole blood (LTOWB), due in part to logistical advantages for the clinical team, and the ability to transfuse RBC, plasma and platelets in a 1:1:1 ratio [10, 11, 12]. The demand for blood products to be used in the prehospital phase of the resuscitation is likely to grow and blood collectors will need to optimize their procedures to ensure sufficient blood is available for patients both in and out of hospital [13].

Currently, most air ambulances in the United Kingdom carry D‐negative RBCs and either dried or thawed plasma. Supply of O D‐negative RBCs is a challenge for most blood operators due to the limited numbers of donors (around 8% blood donors in the United Kingdom and United States are Group O D‐negative). While in England demand for RBCs has fallen by 24% since 2014, this is not mirrored by a similar decrease in demand for O D‐negative RBCs, as noted by other blood collectors [14]. Currently the demand for group O D‐negative RBCs is 14% of total RBCs in England, many issued as the next best alternative when those of RhcDe phenotype are unavailable for the treatment of sickle cell disease [15]. The disparity between demand for group O‐negative RBCs and the proportion of such donors in the population necessitates significant recruitment activity to enrich the donor pool to meet demand [16]. There is concern regarding the long‐term sustainability of supplying group O D‐negative RBCs, especially if the demand for prehospital and treatment of sickle cell disease transfusion increases. Due to limited availability, group O D‐positive RBCs are sometimes used to resuscitate bleeding trauma patients especially in the prehospital phase of the resuscitation in some jurisdictions [17]. This balance of the risk of D‐alloimmunization against providing early balanced resuscitation to injured patients is a particular consideration in relation to the use of LTOWB, which has a shorter shelf life than additive solution containing RBCs, may result in more wastage of the product, but may be more efficacious than current prehospital standard of care in England, that is, RBCs and plasma.

The risk of significant harm due to transfusion of D‐positive blood to bleeding trauma patients of unknown D group is likely smaller than historically thought since (a) 75% to 80% of trauma patients are male [18, 19], (b) the proportion of trauma patients that become D‐alloimmunized is lower than values for healthy individuals [20, 21] and (c) improvements in the diagnosis and treatment of HDFN have markedly reduced the foetal mortality rate where access to modern foetal‐maternal care is available [22]. The aim of this paper was to model the risks of harm from transfusing group O D‐positive RBC components to trauma recipients in England who are bleeding in the prehospital setting.

## MATERIALS AND METHODS

### Model of sequence of events required to cause harm and definition of harms

WE calculated the probability of causing immediate and future harm to a D‐negative patient resuscitated with D‐positive RBCs in the prehospital setting. The harms modelled are the same for RBCs or LTOWB, and therefore, apply to either. We modelled three specific harms:

1. Haemolytic transfusion reaction (HTR) associated with index transfusion of D‐positive blood
  Major morbidity or mortality due to an HTR caused by pre‐existing anti‐D in the patient reacting with D‐positive red cells that were transfused during haemorrhagic trauma resuscitation. This may be acute or delayed and excludes hyperhaemolysis and ABO incompatibility. Major morbidity was defined as per the Serious Hazards of Transfusion Scheme as: Intensive care or high dependency admission and/or ventilation, renal dialysis and/or renal impairment, evidence of acute intravascular haemolysis (e.g., haemoglobinaemia or severe haemoglobinuria) and life‐threatening acute reaction requiring immediate medical intervention, reaction resulting in a low or high haemoglobin level of a degree sufficient to cause risk to life [23].
2. HTR associated with future transfusion of D‐positive blood
  Major morbidity or mortality due to an HTR to a future D‐positive transfusion caused by the anti‐D that was formed following the transfusion of the index D‐positive RBCs during the haemorrhagic trauma resuscitation.
3. HDFN
  Death or lifelong disability of a foetus or child in a future pregnancy caused by HDFN, following maternal anti‐D alloimmunization following the index transfusion of D‐positive RBCs during the haemorrhagic trauma resuscitation.

**FIGURE 1:**
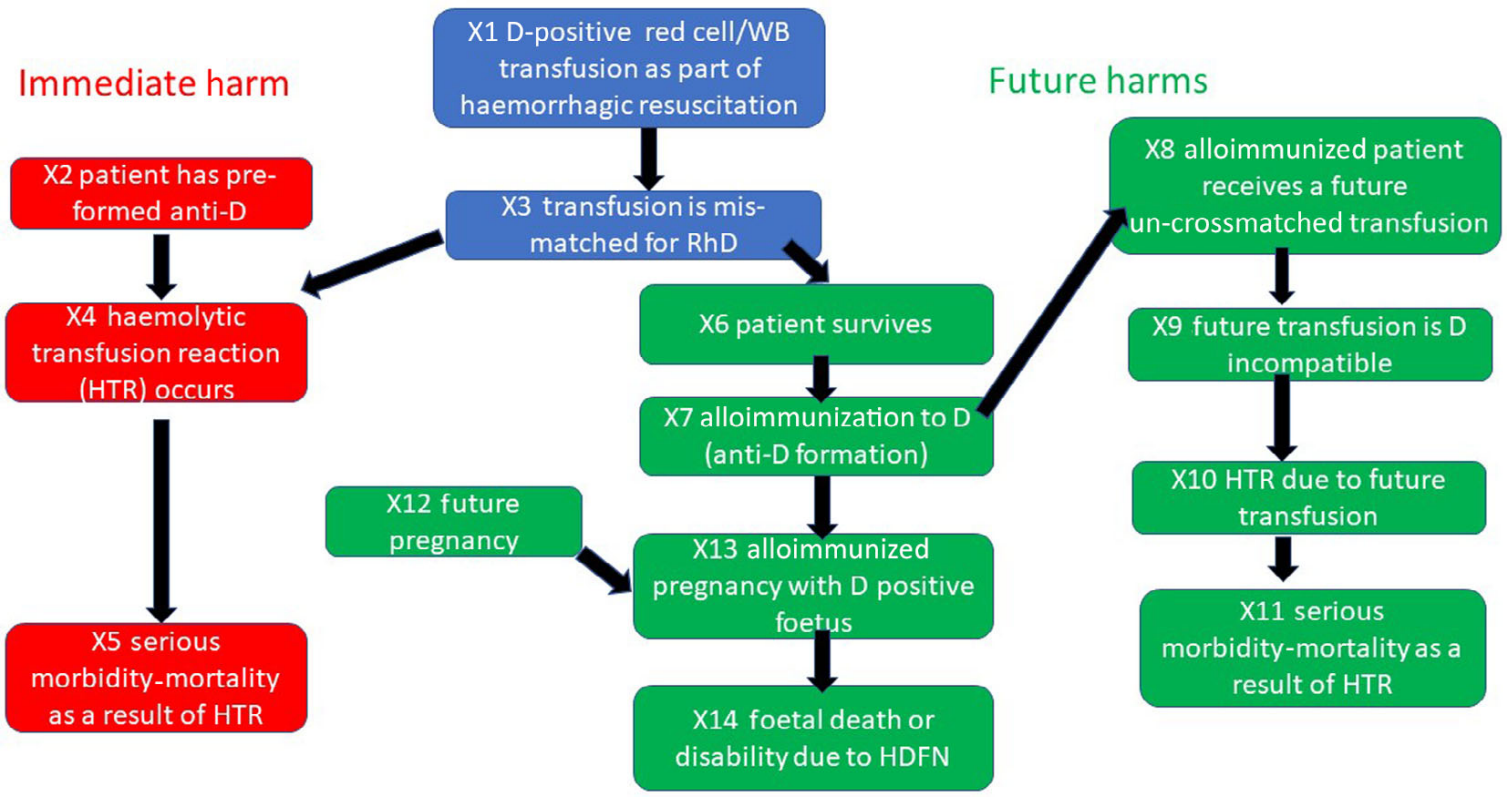
Sequence of events required to cause harm from the transfusion of D‐positive red cells in the prehospital setting. HTR, haemolytic transfusion reaction; WB, whole blood

These harmful events are dependent on the occurrence of a chain of contingent conditions and possibilities, for example, for an HTR to occur due to the index D‐positive transfusion, the patient must have pre‐existing anti‐D. An estimate of the risk of resultant harm can thus be made from estimates of the probabilities of the necessary pre‐requisites in the chain for harm to have occurred. Figure 1 presents the chain of events that are necessary for alloimmunization to occur, and for this alloimmunization to result in an HTR to the index transfusion of D‐positive blood, future HTR or HDFN.

For the model, we assessed two patient populations: (a) all trauma patients of any sex or D type who require haemorrhagic resuscitation with D‐positive RBCs or LTOWB components in the prehospital setting, and (b) the subset of patients who are D‐negative females of childbearing potential (CBP, <50 years of age). The latter analysis was included to model the probability of causing harm to the patient population considered to be most at risk.

### Model inputs and key assumptions used

We modelled the chain of upstream events that are necessary for the various harms to occur as a probabilistic graphical model (Figure 1). For each of the events (X1‐14) that must happen for one of the three harms in Figure 1 to occur, we can estimate the expected number of events seen per recipient assuming the upstream events have occurred (model inputs N1‐14, Table S1). The model inputs are specified in the form of probability distributions, to reflect the level of uncertainty regarding the true value of the input. Where possible, model inputs used published data from the United Kingdom, or if unavailable then published information from the literature was used as described in Table S1.

### Estimation of risk of harm using Monte Carlo simulation

The model was calculated by Monte Carlo simulation using the probability distributions assigned as model inputs. The output allows the determination of credibility intervals (CIs) for the expected number of harmful events. The model was calculated over 1000 iterations, where a random value from the model input distribution is assigned to each event for each iteration. By repeating the process over multiple iterations, the frequency histogram for the event of interest converges to the probability distribution for the event and was used to calculate an estimated mean value and CI. The final expectation was expressed in a reciprocal form (number of transfusions expected to cause one harmful event). Calculations were performed using Wolfram Mathematica 12.0. Further details of the model and its calculation are given in Supplementary information.

In addition, for D‐negative females under 50, we calculated the percentage reduction in 30‐day mortality associated with the use of LTOWB (in terms of life‐years gained) that would be equal to life years lost due to HDFN in future arising from the use of D‐positive LTOWB for 10,000 bootstrap samples from the Monte Carlo simulation results. The survival data described above was used to calculate the probability that life‐years gained would exceed life years lost if the percentage improvement in trauma survival was above a given level compared to current standard of care. For life years lost due to HDFN, we equated either permanent disability or death as 83 life years lost based on current data for UK life expectancy at birth.

### Sensitivity analysis

A sensitivity analysis was undertaken to assess how the level of uncertainty in the individual model input assumptions contributes to the overall uncertainty in the estimate of harm. This sensitivity analysis aimed to help prioritize future work to refine the risk estimate. Two complementary approaches were used. First, scatterplot analysis was used to demonstrate the relationship between the final estimate and individual input factors used in the Monte Carlo simulation. Second, a variance‐based sensitivity analysis whereby the sensitivity index (*S*_*i*_) for model input *N*_*i*_ represents the average proportional reduction in the variance of the final risk estimate if model input *Ni* could be fixed at an exact value [24]. Details of calculations are provided in Supplementary information.

## RESULTS

### Overall estimate of risk for all trauma recipients of D‐positive RBCs

Results of the model showed that for every 14,000 D‐positive transfusions that were administered to all trauma patients of any sex and age, one of the three specific harms (95% CI 5600–42,000) would occur. The frequency of occurrence of the three specific harms is shown in Table 1.

**TABLE 1:** Estimation of risk of harm from transfusion of D‐positive red blood cells or low‐titre group O whole blood in the prehospital treatment of major haemorrhage using model inputs and Monte Carlo simulation

Harm	All recipients One event every × transfusions (95% CI)	D‐negative females of childbearing potential One event every × transfusions (95% CI)
Major morbidity or mortality due to HTR from index D‐positive transfusion	2.7 × 10^4^ (7.6 × 10^3^–3.4 × 10^5^)	6.6 × 10^3^ (1.8 × 10^3^–9.2 × 10^4^)
Major morbidity or mortality due to future HTR	8.5 × 10^5^ (1.8 × 10^5^–2.1 × 10^7^)	1.4 × 10^5^ (3.1 × 10^4^–3.7 × 10^6^)
Foetal death or permanent disability due to anti‐D HDFN in future pregnancy	2.9 × 10^4^ (1.2 × 10^4^–1.2 × 10^5^)	570 (260–2300)
Any of above three harms	1.4 × 10^4^ (5.6 × 10^3^–4.2 × 10^4^)	520 (250–1700)

Abbreviations: CI, credibility interval; HDFN, haemolytic disease of the foetus and newborn; HTR, haemolytic transfusion reaction.

### Risk estimate for D‐negative females <50 years of age

For the highest risk subgroup (D‐negative females under age 50), the model showed that for every 520 D‐positive transfusions, one of any of the three specific harms would occur (CI 250–1700).

The main risk in this population was that of HDFN, where for every 570 transfusions, one foetal death or disability would occur (CI 260–2300), which is higher than the risk of the other two specific harms (contemporary and future HTR).

The data from Table 1 were used to calculate the expected length of time before a specific harm would be expected to occur if a policy of using group O‐positive RBCs for prehospital transfusion is introduced universally across England. There are no UK data to estimate how often un‐crossmatched blood is transfused prehospital for trauma patients, so we used the data from Stanworth et al. [18] as a surrogate, who reported the overall incidence of major haemorrhage in trauma as 83 per million patients per year (defined as receiving at least 4 RBC units in the first 24‐h of hospital admission) in England, using 2020 population figures for England of 67 million. The results of the modelling demonstrated that the number of years required for any of the three specific harms to occur is 2.5 (CI 1.0–7.5) for all recipients, and 5.2 (CI 2.5–17.3) for D‐negative females of CBP (Table 2). HDFN would be expected to occur every 5.7 years in the latter group. Based on the assumptions and data used in this model, in 95% of the simulations, a survival increase of at least 1.0% from the use of LTOWB compared to current standard of care in the United Kingdom, would lead to life years gained exceeding life years lost due to HDFN in the future if D‐positive whole blood was transfused to D‐negative females under 50. Thus, we can be 95% confident that if the trauma survival improvement was greater than 1%, then life‐years gained associated with the use of LTOWB would exceed years lost due to HDFN for the latter group of patients.

**TABLE 2:** Predicted number of years to observation of harm in England based on model if a change in policy to use D‐positive red blood cells or low‐titre group O whole blood was implemented

	All recipients	D‐negative females of childbearing potential
Prehospital transfusions per year	5561	100
Years to one HTR major morbidity or death due to index D‐positive transfusion	4.8 (1.4–61)	66 (18–910)
Years to one future HTR major morbidity or death	150 (35–3700)	1400 (310–37,000)
Years to one future anti‐D HDFN death or disability	5.2 (2.2–21.6)	5.7 (2.6–22.5)
Years to any of three above harms	2.5 (1.0–7.5)	5.2 (2.5–17.3)

Abbreviations: HDFN, haemolytic disease of the foetus and newborn; HTR, haemolytic transfusion reaction.

The risk of an HTR due to an index D‐positive transfusion was 1:27,000 transfusions for all recipients and 1:6600 for D‐negative females under 50; this translates to one event occurring every 4 years and 65 years, respectively, in England.

Sensitivity analysis of the overall risk estimate to individual model inputs is shown in Figures S4 and S5. These Supplementary data show that for unselected recipients, uncertainty in the risk of HTRs dominates the uncertainty of the total risk. In contrast, for D‐negative recipients under 50, the uncertainty of the total risk is dominated by uncertainty regarding the alloimmunization risk.

## DISCUSSION

We modelled the three main risks associated with the transfusion of D‐positive RBCs to trauma patients of unknown D type, as would be the case when transfusions are administered in the prehospital and early in‐hospital phases of the resuscitation. In general, the risks of acute or future HTR and HDFN are low, especially when compared to the benefits of providing transfusions early in the resuscitation. The risk of HTR from future transfusions is several orders of magnitude lower than that of HDFN.

The most feared sequelae of transfusing D‐positive RBCs to patients who are later found to be D‐negative is foetal demise or the requirement for intensive, and often invasive, ante‐ and post‐partum management of HDFN. These data predict that one foetal death or disability due to HDFN will occur for every 500 transfusions to females of CBP (once every 5 years in practice in England), if exclusively D‐positive transfusions are provided in the prehospital setting to all patients. Our modelling assumed that D‐positive blood would be exclusively transfused instead of RBC negative units, that is, the scenario with the highest potential for causing anti‐D‐related adverse events. If D‐positive blood is provided as a 50:50 mix with D negative units, or only if D‐negative units are unavailable, then these risks would be lower. At one large American trauma centre [25], the authors calculated that the overall risk of experiencing HDFN would be 1.2/100 transfused D‐negative females, or approximately 1 HDFN case every 20 years. At another American trauma centre, it was estimated that it would take 250 years for 3–30 females, to become D‐alloimmunized due to the relative infrequency at which these females are transfused at that centre [17]. During this time, 500 females of CBP would die from haemorrhage if prehospital transfusions were not available. We predicted that a case of HDFN would be expected to occur in England more frequently than at these American centres, likely due to the total higher number of females of CBP, approximately 20‐fold that would be transfused throughout the country of England compared to the number transfused at a few regional trauma centres.

The strength of our study is that we have, for the first time, modelled the risk of a national policy change to the use of group O D‐positive blood in the prehospital setting, based on the best available data and applied accepted mathematical modelling to illustrate the uncertainty in these estimates. This modelling permits the estimation of a credible range of frequencies for the potential risk, taking account of the uncertainty in the assumptions in parallel, allowing the use of conservative risk estimates in policy making. There is a high level of uncertainty regarding some of the underlying assumptions due to limited data on the frequency of occurrence of many of the events that were modelled. For example, the uncertainty surrounding the frequency of the overall risk of the three specific harms for females of CBP is dominated by uncertainty over the risk of alloimmunization. It can be seen from the factor analysis scatterplot that if the risk of immunization was 10%, then we would expect to see a specific harm every 7–17 years, whereas if the alloimmunization risk was 40%, we would expect to see a harmful event every 1–5 years. Nevertheless, the effect of uncertainty in the other variables means that the variance of the final risk estimate would only be reduced by 11% even if the value for risk of alloimmunization were certain. In other words, expending effort in performing studies to firm up the alloimmunization risk assumption would not have a large impact on final risk estimates.

While a rate of having any one of the three studied specific harms occurring once for every 14 × 10^3^ D‐positive transfusions in the overall population is nearly the same as the reported rate of transfusion‐associated circulatory overload (1:25 × 10^3^ transfusions) [23], the rate of anti‐D‐mediated HDFN among D‐negative females of CBP is much higher at (one case of HDFN per 52 × 10^1^ D‐positive transfusions) in the prehospital setting. However, the survival benefits of early transfusion in trauma resuscitation must be balanced against these risk estimates. Based on the data used in our model, life years gained from a 1% reduction in mortality compared to current standard of care would be expected to exceed life years lost due to the risk of HDFN for D‐negative females under 50 years of age. This value would be lower for all recipients of LTOWB prehospital since any survival benefit would be seen across the entire population with little increased risk of HDFN compared to that for D‐negative females under 50. Compared to using only saline as the resuscitation fluid, several studies have demonstrated significantly better survival when blood products are administered during the patient's transport to the hospital [6, 7, 8, 9]. Most of the air ambulance services in England already provide D‐negative RBCs to their patients as well as some form of plasma, thus not conferring a risk of D‐alloimmunization. So why change the current standard of care? Although the UK blood services have managed to supply D‐negative RBC to prehospital services, if demand rises, further demand for D‐negative RBCs may outstrip supply, and if this occurs, the resuscitation options would be either to revert to saline with its inherent risks and little benefit to the injured recipient [26], or to transfuse D‐positive RBCs or LTOWB. Risk‐assessments like this one, are vital to estimating the risks of harmful events associated with transfusion of D‐positive units to D negative recipients. These need to be balanced against the benefits of either red cell or whole blood transfusion in the prehospital setting, which will depend upon the standard of care and availability of D‐negative units in each jurisdiction. It would also be possible to mitigate the risk of HDFN by using D‐positive blood only for males, and D‐negative LTOWB for women of CBP. However, in practice, prehospital teams may find it challenging to store, transport and provide multiple types of blood.

The current philosophy of resuscitating trauma patients early with blood transfusion is shifting towards providing a balanced resuscitation that includes platelets in addition to RBCs and plasma, similar to whole blood [10, 11, 12]. Multiple donor qualifications are required in order to be eligible to donate LTOWB, which makes it challenging for blood providers to supply RhD‐negative LTOWB to all trauma patients. As a result of these supply issues, the use of D‐positive LTOWB and RBCs for injured patients early in their resuscitation is becoming a common practice at least in the United States; in an international survey of LTOWB practice (mainly from the United States), 27% of the respondents reported using D‐positive LTOWB for females of any age [27], while a survey of American Level 1 trauma centres revealed that 51% would administer a D‐positive LTOWB unit to a female of CBP whose D‐type was unknown in a bleeding emergency [28].

Based on these risk estimates, a survival benefit of 1% or greater from transfusing red cells compared with no transfusion or whole blood versus red cells in the prehospital setting, outweigh the risks of harmful events associated with transfusion of D‐positive units to D‐negative recipients. In addition, reallocation of D‐negative RBCs away from prehospital use would facilitate the provision of these scarce RBCs to patients who are already D‐alloimmunized, and patients who require antigen‐matched RBC such as sickle cell disease patients for whom alloimmunization could pose dire long term health consequences. A survey of transfusion and trauma services directors at the 30 largest children's specialty hospitals in the United States revealed some reluctance to enroll injured girls of unknown D‐type in a study of D‐positive LTOWB transfusion versus component therapy, consistent with the traditional practice of providing D‐negative blood components in this clinical setting [29]. However, in a survey of staff at a large American university, 90% of the respondents who were females of CBP indicated that they would accept a lifesaving transfusion even with the knowledge that it could harm future pregnancies [30]. Further work is needed to understand the views of patients and the general public as to whether these risk are acceptable, so that their views can be taken into account when determining policy. In addition, protocols and surveillance for follow‐up of D‐negative females of CBP who receive D‐positive whole blood or red cells are required.

## CONFLICT OF INTEREST

R.C. is a member of Terumo BCT's Physician Advisory Board, L.G. and M.Y. have received expenses for speaking at conferences sponsored by Terumo BCT.

## Supporting information


**Data S1.** Supporting information.
