# Supplementary material for: Estimating the risks of prehospital transfusion of D‐positive whole blood to trauma patients who are bleeding in England
Source: Vox Sang. 2022 Jan 12;117(5):701–7. doi: 10.1111/vox.13249 (PMC9306525; doi:10.1111/vox.13249)
Supplement: Supplementary file 1 — Data S1. Supporting information. [file VOX-117-701-s001.docx]

**Supplementary methods**

*1. Probablistic Graphical Model formulation*

We constructed a probabilistic graphical model of the chain of events necessary for harm to occur as follows:

The events of interest are denoted X_1_, X_2_...X_k_

A graph G:={N_i_,E_ij_}was defined to represent the relationship between the events of interest:

A node Ni was defined in the graph associated with each event Xi.

A directed edge (or arrow) E_ij_ was defined between node N_i_ and N_j_ if event X_j_ is dependent on event X_i_ occurring.

The set of parents of a node N_i_ is defined as the set of nodes which have arrows pointing to N_i_:

π_i_ :={N_i_: E_xi_ ≠ 0}.

The weight assigned to each node represents the conditional expectation of the associated event given that exactly one of each parente occurs:

1.1 w(N_i_)= Exp(X_i_ | E_xi_=1 for all x: N_x_ є π(N_i_) )

w(N_i_)= Exp(X_i_) if π(N_i_) is empty.

This will be equivalent to the conditional probability in many cases here, since the incoming events are singular (single recipient or transfusion event).

An estimate for the range of credible values for the conditional expectation of each event X_i_ was assigned to each node, in the form of a probability density function f_i_(x_i_):

1.2 w(N_i_) ~ f_i_(x_i_)

The set of probability density functions assigned are referred to as the *model inputs*. The rationale for the choice of individual model inputs is outlined in supplementary section 2 below.

The weight assigned to the outgoing edge from each node N_i_  represents the expected value for event X_i._  This is calculated recursively as the product of the conditional expectation assigned to node N_i_ and of the weight of all incoming edges:

1.3 w(E_ix_)= w(N_i_). $\prod_{jє\pi(Ni)}$w(E_j,i_)  for all arrows exiting from Ni to node x

A terminal node with no outputs and node weight 1 was defined to “collect” the posterior expectation of events with no children.

*Calculation by Monte Carlo Simulation*

For each iteration of the Monte Carlo simulation , a randomly sampled value from the associated model input distribution (as in 1.2) was assigned to each node:

1.4 w(N_i_):= F_i_^-1^(u)

where u is a random real uniformly distributed in [0,1] and   F_i_^-1^ (u) is the inverse cumulative function of f_i_(x_i_) ie  F_i_^-1^ (u) := P(x_i_<u)

The expectation for each event was then calculated and assigned to the outgoing edges, starting from nodes with no parents:

1.5 w(E_ix_)= w(N_i_). $\prod_{jє\pi(Ni)}$E_j,i_  for all arrows exiting from Ni to node x

1000 iterations of the Monte-Carlo simulation were performed, repeating steps 1.4 and 1.5. 1000 iterations was empirically found to give stable results for the principal harms, to 2 significant figures.

Then the estimate for the cumulative probability distribution of Xi given N iterations is the frequency histogram of the values of Xi calculated in each iteration:

1.6 P(X_i_<t) =[$\sum_{n=1}^{N}$ #(w_n_(E_ij_)<t)  ] /N

where #(w_n_(E_ij_)<t)  = 1 if w(E_ij_)<t in iteration n.

*2. Rationale for form of assigned distributions*:

*General principles*

A beta distribution was assigned for the conditional expectation of an event if it is estimated from a single study with a known number of participants. Beta distributions were used in preference to normal approximations to the binomial distribution because the number of study participants in the available evidence was often small.

A triangular distribution was used to combine the results from multiple studies, with the peak of the distribution selected as the combined average from all studies combined, and the extreme values as the highest and lowest 95% confidence interval bounds.

A uniform distribution is assigned over a credible range where an estimate is made based on data which is only weakly analogous to the event of interest. A uniform distribution is also assigned where the conditional expectation is derived from an estimate in the literature which does not attempt to express the uncertainty of the estimate- in this case the uniform distribution is arbitrarily assigned a 10% range either side of the estimate.

A normal distribution was assigned where estimates are derived from population statistics, or by simulation.

*Choice of individual model inputs*

N1: Expected proportion of individuals who receive a pre-hospital transfusion who are transfused D positive blood during the prehospital transfusion: Assigned probability of 1, by definition as the standard of care being modelled

N2: Expected proportion of recipients with preformed anti-D before receiving D-positive RBCs during prehospital trauma: Assigned Uniform [0.016,0.03 ] based on a range of studies estimating the prevalence of anti D in pre transfusion sample as between 1.6% and 3% , and Uniform [0.01,0.02] for D negative females under 50.^31^

N3: Expected proportion of index transfusions mismatched for D (D-positive RBC transfused to D-negative recipient), given that a D positive transfusion has been transfused. Assigned Uniform [0.15+/-0.015] or 1 for D negative females under 50.  Equivalent here to the probability that the recipient is D negative.^31^

N4: Expected proportion of recipents suffering an HTR given that a D positive index transfusion is transfused to a recipient with anti-D. Assigned Beta[2,6] based on1 transfusion reaction in 7 recipients who received incompatible blood  in a retrospective review of 262 patients with alloantibodies who received uncrossmatched transfusion.^32^

N5: Expected proportion of recipients suffering major morbidity or mortality following aa haemolytic transfusion reaction, given that an HTR has occurred. Assigned Beta[3,65] based on haemovigilance data where 2/66 HTR cases were classified as “severe”.^23^

N6: Expected proportion of recipients who have received a prehospital transfusion who survive the episode of trauma. Assigned Beta [390,404] for all recipients and Beta [49,48] for D negative females under 50, based on 30-day survival data from the UK prehospital transfusion dataset.

N7 Expected proportion of recipients developing D-alloimmunisation following the administration of an D-positive RBC to an D-negative trauma recipient. Assigned a Triangular distribution with peak value 0.22 and limits 0.038 to 0.53, based on published studies with a combined 140/627 patients being immunised.^33–36^ The lower 95% confidence interval of the study reporting the lowest incidence predicted an immunisation rate of above 3.8% immunisation rate, and the upper 95% confidence interval of the study showing the highest incidence predicted an immunisation rate below 53%.

N8: Expected proportion of alloimmunised trauma survivors who receive a future un-crossmatched RBC transfusion  Stanworth estimated the incidence of major trauma haemorrhage as 83/million per year, which is used as a surrogate estimate for the incidence of out of hospital transfusion.^18^  The average life expectancy for a trauma recipient is 44 years based on the age and sex of trauma recipients and the 2018 Life Tables from the Office of National Statistics [see commentary below]. This suggests a lifetime probability of a further major haemorrhage in the order of [1/100 to 1/1000]. Assigned Uniform[0.001,001] in view of the difficulty of estimation.

N9: Expected proportion of future uncrossmatched  transfusion which are D positive given that an uncrossmatched transfusion has been given. Assigned probability of 1,  as a “worst case estimate”, based on the premise that the policy of D positive transfusion for emergency transfusion is implemented in the future.

N10: Expected proportion of future transfusions of D posititve blood to a recipient with anti D which cause a HTR. Assigned Beta[2,7] as in N4 above^32^

N11:  Expected proportion of future haemolytic transfusion reactions which result in severe morbidity or mortality. Assigned Beta[3,65] as in N5 above.

N12: Expected number of future pregnancies for a recipient of prehospital transfusion who survived initial trauma. Assigned Normal [0.1,0.018] for all recipients and Normal [0.81,.09] for D negative females under 50. See supplementary section 3 below.

N13: Expected proportion of alloimmunised pregnancies carrying a D positive fetus:  Assigned Uniform [0.6 +- 0.06%], using the Hardy-Weinberg equilibrium estimate of inheriting a D positive allele from the father, given a population D negative frequency of 0.15.^31^

N14: Expected proportion of alloimmunised pregnancies carrying a D positive fetus which result in fetal death or permanent disability due to anti D HDFN. Assigned Normal [0.027, 0.0065]. See supplementary section 4 below.

*3. Estimation of life expectancy and future children for trauma recipients.*

Estimation of life expectancy and future children for trauma survivors (used to estimate X6 and X8).

Demographics of the age and sex distribution (supplementary Figure 1) for trauma patients presenting with major bleeding and who required prehospital blood transfusion (total of 671 patients) was obtained from a recent Red Cell and Plasma study that prospectively collected clinical data from all trauma patients who required blood transfusion from eight pre-hospital services in England between October 2018 to October 2020 (personal communication, Dr Laura Green, 2021). The aim of this study was to compare the clinical outcomes of trauma patients who were transfuse a combined blood component (red cell and plasma) in London in pre-hospital setting versus those who received RBC and thawed plasma transfusion (or lyophilised plasma) outside London (seven pre-hospital services). In England there are 18 air ambulance services in total of which the eight that participated in the above study are amongst the biggest users of blood in pre-hospital environment, and thus we believe this sample size is representative of the UK services.

The expected number of future children for a trauma recipient was estimated using birth rate data grouped by maternal age and the age/sex distribution of trauma survivors from the data above. Birth rate data was obtained from the UK Office of National Statistics (ONS) (supplementary Figure 2). The average life expectancy for a trauma recipient was estimated using the 2018 Life Tables from the ONS, which provide actuarial life expectancy given the subject’s current age and sex (supplementary Figure 3). Uncertainty for the estimate was obtained by bootstrapping 1000 iterations from the UK pre-hospital datasets. Further details of these estimates are given in the supplementary material

Define

3.1 F(age,sex) = sex* $\int_{t=age}^{45} B(t) dt$

where

sex=0 if recipient is male, sex= 1 if recipient is female,

B(t) is the birth rate (average births per individual per year for an individual of age t)

B(t) was approximated by linear interpolation from birth rate data from the Office of National Statistics (ONS) which is grouped by integer ages. The sum was taken up to age 45 as the ONS does not publish data above this age (in other words considering the birth rate to be negligible after age 45)

Then the expected number of future children from a sample of N subjects with the demographics of the *i*th subject given as (age_i_, sex_i_) was calculated as

3.2 ($\sum_{i=1}^{N}$F(age_i_, sex_i_) )/N

The average life expectancy for a trauma recipient was estimated using the 2018 Life Tables from the ONS, which provide actuarial life expectancy given the subject’s current age and sex.

The average life expectancy for a trauma recipient was thus calculated as

3.3 ($\sum_{i=1}^{N}$L(age_i_, sex_i_) )/N

where L(age, sex) is the life expectancy for an individual of given age and sex.

*4. Estimation of the risk of death or permanent disability due to HDFN.*

This was estimated as

4.1 P4=P1 x (1-(1-P2) x (1-P3) )

where

P1=probability that a pregnancy immunised with anti D and carrying a D positive fetus needs an intrauterine transfusion (IUT) for anti-D HDFN. Estimated from 52/260 D-immunised pregnancies needing IUT,^37^ assuming that only D positive pregnancies needed IUT in the study, and assuming that the proportion of D positive fetuses is 0.6.

P2=probability that a pregnancy needing IUT due to anti-D HDFN results in fetal death. Estimated from death rate of 4/111 in IUTs performed for HDFN between 2011 and 2016.^22^

P3=probability that a live-born infant who needed IUT for anti D HDFN suffers long term disability. Estimated from the finding of neurodevelopmental disorders in 14/291 infants who received IUT for HDFN.^38^

P4=probability that an pregnancy immunised with anti D and carrying a D positive fetus results in death or disability, based on the assumption that all cases of death or disability were identified as “needing an IUT”.

A probability distribution for P4 was obtained by Monte Carlo simulation of formula 4.1 using samples from the normal approximation to the binomial distribution for P1-P3 for 1000 iterations. The resulting frequency histogram was well approximated by a normal distribution [p<0.1x10^-4^ , Kolmogorov-Smirnov test for goodness of fit], which was used as the input N14

*5. Sensitivity analysis.*

The sensitivity of the final estimate of harm to variability in the individual input variables was visualised by plotting factor analysis scatterplots of the final risk estimate against the value of the input variable for each iteration of the Monte Carlo simulation [Supplementary figure 4].

Additionally, a variance-based sensitivity index was calculated as outlined below. Informally, the sensitivity index describes the average proportion reduction if the exact value of the input factor was known.

Let Y be the final risk estimate, dependent on model inputs N_1_.... N_k_

The Sensitivity Index for model input i is defined as

5.1 S_i_ := [Var(Y) -Var(Exp(Y|N_i_) ] / Var (Y)

The sensitivity index was calculated from the Monte Carlo simulation outputs using the method described in Saltelli (2008):

5.2 S_i_=1- [ <**y_A_** , **y_Ci_**> - mean(**y_A_**)^2 ] / [ <**y_A_** , **y_A_**> - mean(**y_A_**)^2 ]

where

**y_A_** is the vector of estimates from Monte Carlo iterations 1...N/2

**y_Ci_** is the vector of estimates recalculated using model inputs from iterations N/2+1 ...N *but replacing input i with the input from iterations 1...N/2.*

and <**a,b>** is the standard vector dot product

**Supplementary results**

Table 1. Summary of model input parameters and assumptions.

|  | Input | Estimate* (Mean with 95% CI) | Rationale | Reference |
| --- | --- | --- | --- | --- |
| N1 | Expected proportion of individuals who receive a pre-hospital transfusion who are transfused D positive blood during the prehospital transfusion. | 1 (1-1) | Modelling the risk of transfusing D positive as standard of care | --- |
| N2 | Expected proportion of recipients with preformed anti-D before receiving D-positive RBCs during prehospital trauma resuscitation | 0.023  (0.016 – 0.030) | Based on a range of studies estimating the prevalence of anti-D in pre transfusion samples between 1.6% - 3%. Estimated as 0.015 (0.01-0.02) for D negative females of CPB. | ^31^ |
| N3 | Expected proportion of index transfusions mis-matched for D (D-positive RBC transfused to D-negative recipient) | 0.15 (0.15 – 0.16) | Equivalent to the probability that the recipient is D negative. Value is set to 1.00 for D negative females of CPB for this analysis. | ^31^ |
| N4 | Expected proportion of recipents suffering an HTR following the index administration of an D positive RBC unit to a recipient with anti-D. | 0.22  (0.032 – 0.53) | 1 HTR amongst 7 recipients who received incompatible RBCs in a retrospective review of 262 patients with alloantibodies who received uncrossmatched transfusions | ^32^ |
| N5 | Expected proportion of recipients suffering major morbidity or mortality following a haemolytic transfusion reaction. | 0.044  (0.0093 – 0.1) | 2/66 cases classified as major morbidity or death | ^23^ |
| N6 | Expected proportion of recipients who have received a prehospital transfusion who survive the episode of trauma. | 0.48 (0.42-.54) | Based on recent UK prehospital data set where 134/280 recipients survived at 30 days. 0.51 (0.41-0.6 for D negative females of CPB for this analysis) | See methods |
| N7 | Expected proportion of recipients developing D-alloimmunisation following the administration of an D-positive RBC to an D-negative recipient | 0.26(0.085-0.47) | Based on 7 published studies with a combined total of 140 /627 patients being immunised | ^33–36^ |
| N8 | Expected proportion of alloimmunised trauma survivors who receive a future un-crossmatched RBC transfusion | 0.0055  (0.0012 – 0.0098) | Equal to the number of years lived having survived the initial trauma times the expected number of uncrossmatched emergency transfusions per year. Incidence of major trauma haemorrhage estimated as 83/million per year. The average life expectancy for a trauma survivors is 45 years based on the age and sex of trauma recipients and the 2018 Life Tables from the Office of National Statistics [see methods section]. | ^18^ |
| N9 | Expected proportion of future un-crossmatched RBC transfusions which are D- positive. | 1 | Assigned probability of 1.00, as a “worst case” estimate, based on the premise that the policy of D-positive transfusion for emergency transfusion is implemented. | Worst case estimate |
| N10 | Expected proportion of future transfusions of D posititve blood to a recipient with anti D which cause a HTR. | 0.22 (0.32 – 0.53) | Same assumptions as X4 | ^32^ |
| N11 | Expected proportion of future haemolytic transfusion reactions which result in severe morbidity or mortality. | 0.044 (0.0093 – 0.1) | Same assumptions as X5 | ^23^ |
| N12 | Expected number of future pregnancies for a recipient of prehospital transfusion. | 0.1 (0.065-.14) | Based on age and sex of trauma survivors from UK data set described in methods, and birth rate data from the Office of National Statistics. See methods section. Estimated value for D negative females of CBP 0.81 (.63-.99) | See supplementary information for calculation of estimate |
| N13 | Expected proportion of D-alloimmunised pregnanciescarrying a D positive fetus | 0.60 (0.54-0.66) | Probability of inheriting a D positive paternal allele given a 15% D negative frequency | ^31^ |
| N14 | Expected proportion of alloimmunised pregnancies carrying a D positive fetus which result in fetal death or permanent disability due to anti D HDFN. | 0.027(0.014-.04) | Based on 52/260 D-immunised pregnancies needing IUT, death rate due to IUT of 4/111 and rate of neurodevelopmental disorders in infants who received IUT for HDN of 14/291. | ^22,37,38^  See supplementary information for calculation of estimate |

The values shown are for all recipients, with variations used for modelling D-negative females of CPB only noted in the rationale. HTR=haemolytic transfusion reaction, IUT= intrauterine transfusion. “Index transfusion” refers to the transfusion given at the time of pre-hospital resuscitation; “future transfusion” refers to a transfusion given in a separate event following completion of the trauma episode.

**Supplementary Figure 1: Age distribution of trauma survivors**

**
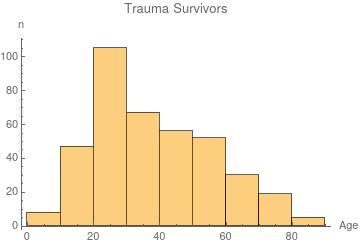
**

**
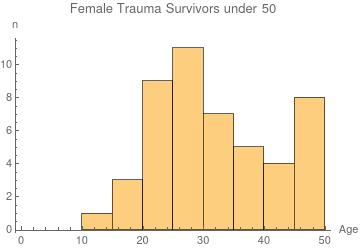
**

**Supplementary Figure 1.** Frequency histograms illustrating the age demographics of trauma survivors from the UK prehospital transfusion dataset.

**Supplementary Figure 2: UK Birth rate**

**
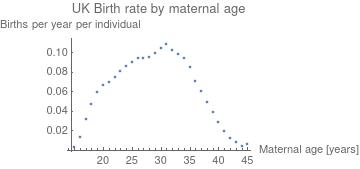
**

**Supplementary Figure 2:**  Illustrating the UK birth rate by maternal age, according to 2018 data from the Office of National Statistics.

**Supplementary Figure 3: UK Life expectancy**


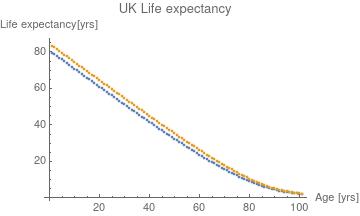


**Supplementary Figure 3:**  Illustrating the UK life expectancy by age, according to 2018 data from the Office of National Statistics. The upper line represents life expectancy for females and the lower line for males.

**Supplementary Figure 4: Factor analysis scatterplots.**

Supplementary figure 4a- all recipients


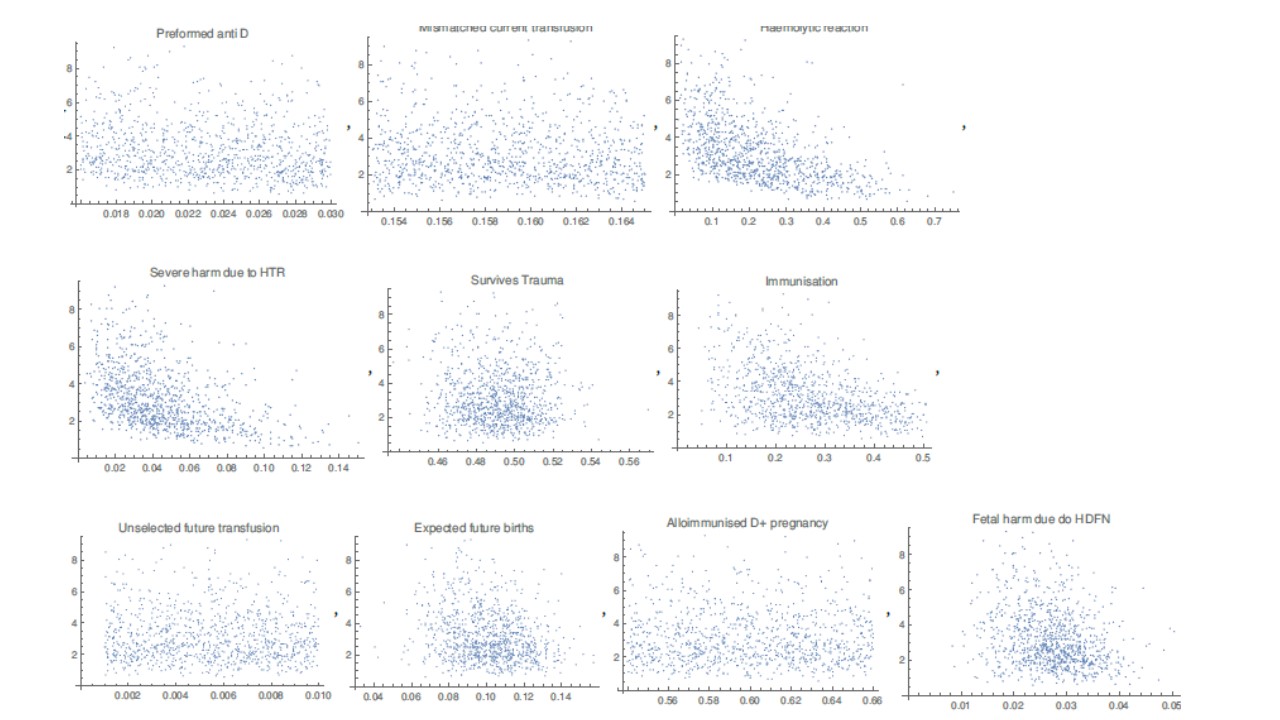


Supplementary Figure 4b- D negative females under 50


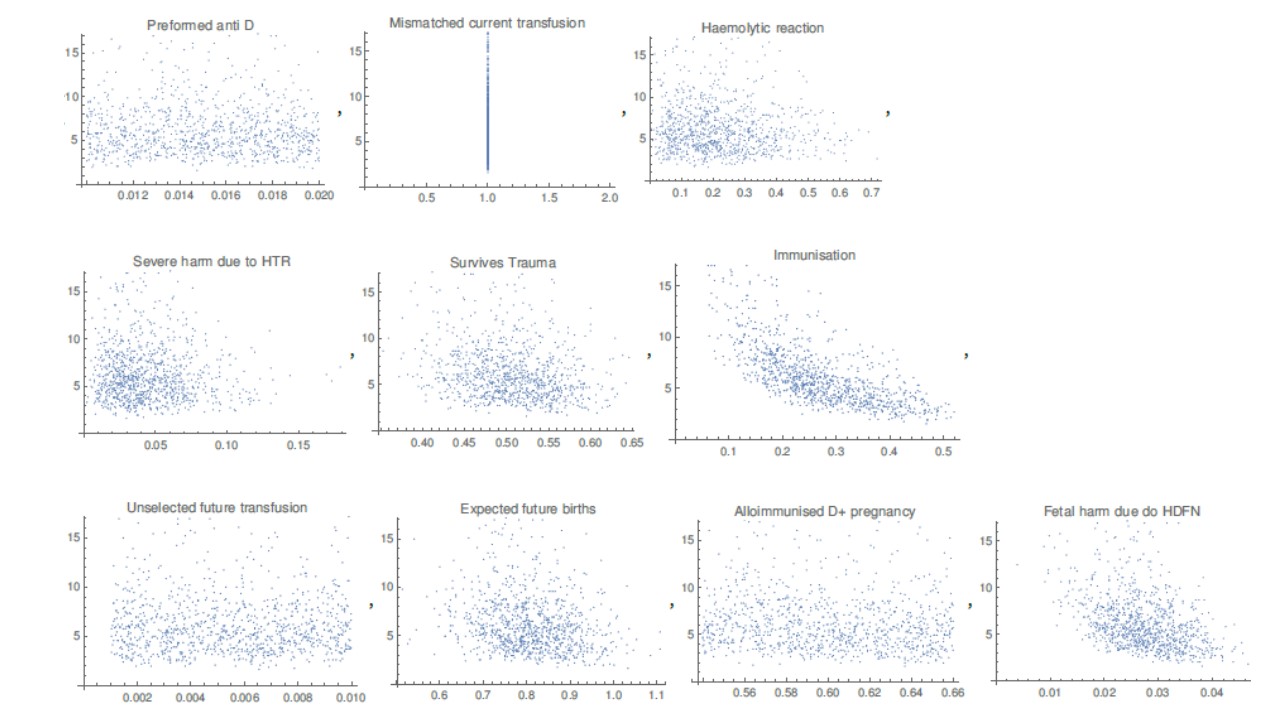


**Supplementary Figure 4. Individual factor analysis.** Scatterplots showing the effect of individual input factors on the overall estimate of risk. Each point represents one iteration of the Monte Carlo simulation. The X value for each point represents the value used for the input for that iteration and the Y value is the expected number of years to see 1 harmful event for that iteration. A flat distribution of points for the scatterplot across the range of an input variable (for example in the scatterplots for the risk of needing a future transfusion) indicates that the variable has little effect on the final estimate of total risk. Estimates of the total risk for particular values of an input variable can also be visualised by looking at the spread of points along a slice through the X axis at the value of interest. For example, the total risk of harm to D negative females under 50 if the alloimmunisation risk was 0.2 can be estimated as one event every 4-16 years, examining the plot at the end of the second row in figure 4b.

**Supplementary Figure 5 Sensitivity Analysis**

Supplementary Figure 5a

**
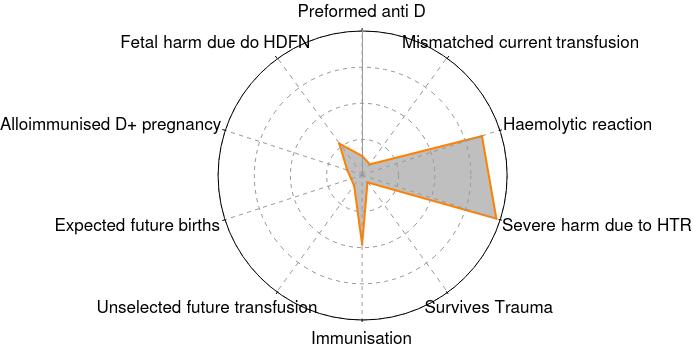
**

Supplementary Figure 5b- D negative females under 50


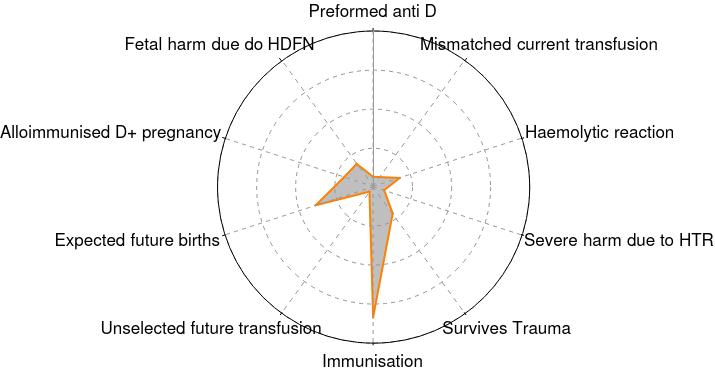


Supplementary Figure 5: Radar plots illustrating the relative contribution of individual impact factors to the overall uncertainty. The radius for each factor is proportional to the square root of the sensitivity index shown in Supplementary table 1 and thus to the reduction in standard deviation if the input factor was known exactly.

**Supplementary Table 1: Sensitivity index.**

Supplementary Table 1a All recipients

| Input | Factor | Sensitivity Index |
| --- | --- | --- |
| N2 | Preformed anti D | 0.0028 |
| N3 | Mismatched current transfusion | 0.0013 |
| N4 | Haemolytic reaction if D positive transfused to recipient with antibodies. | 0.12 |
| N5 | Severe harm due to HTR | 0.15 |
| N6 | Trauma survival | 0.00064 |
| N7 | Immunisation | 0.037 |
| N8 | Unselected future transfusion | 0.0014 |
| N12 | Expected future births | 0.0010 |
| N13 | D+ pregnancy | 0.0019 |
| N14 | Fetal harm due do HDFN | 0.012 |

Supplementary Table 1b D negative females under 50

| Input | Factor | Sensitivity Index |
| --- | --- | --- |
| N2 | Preformed anti D | 0.00063 |
| N3 | Mismatched current transfusion | 0 |
| N4 | Haemolytic reaction if D positive transfused to recipient with antibodies. | 0.0051 |
| N5 | Severe harm due to HTR | 0.00088 |
| N6 | Trauma survival | 0.0073 |
| N7 | Immunisation | 0.11 |
| N8 | Unselected future transfusion | 0.00025 |
| N12 | Expected future births | 0.024 |
| N13 | D+ pregnancy | 0.0061 |
| N14 | Fetal harm due do HDFN | 0.0052 |

Supplementary Table 1: Sensitivity index (Si) for the sensitivity of the total risk estimate to individual input factors. Si represents the expected reduction in variance of the risk of harm if the value of the individual factor was known exactly. Thus a low sensitivity index indicates that further work to improve the estimate of the factor would have little impact on the revised risk estimate.
